# Spontaneous inactivating p53 mutations and the “selfish cell”

**DOI:** 10.18632/aging.100294

**Published:** 2011-03-09

**Authors:** Steven M. Sorscher, Aubrey E. Hill, Roger Belizaire, Eric J. Sorscher

**Affiliations:** ^1^ Washington University, St. Louis, MO 63110, USA; ^2^ University of Alabama, Birmingham, Al 35294, USA

Odell *et al.* reported in Aging that murine embryo fibroblasts (MEFs) undergo p53 mutations and subsequent immortalizations in culture. This “release from cell cycle arrest” occurs in murine fibroblasts with a humanized or murine WT p53 [1]. The authors also noted that the cultured cells had frequently sustained a mutation matching a human tumor p53 mutation. Jang, *et al*. also reported spontaneous “immortalization and tumorigenic transformation” of human keratinocytes associated with p53 inactivating mutations [2].

Odell's remarkable findings are of great interest to us and support a recently proposed hypothesis for tumorigenesis. We and others have proposed that cells seek to survive, in a Darwinian sense [3, 4, 5]. Specifically we have presented evidence to suggest that while WT p53 might primarily function as the “guardian of the genome” (inducing cells to undergo apoptosis or cell cycle arrest under stress), the WT p53 gene sequence is evolutionarily maintained such that it is susceptible to inactivating mutations and consequent cell survival (with the untoward result of tumorigenesis). The facts that these mutations occur spontaneously and frequently are the exact mutations seen in human cancers lends key support to this notion. Taken together, spontaneous mutations allowing cell survival and tumorigenesis might be referred to as the “Selfish Cell Theory” in recognition of Dawkin's The Selfish Gene published in 1976.
